# *MTDH* genetic variants in colorectal cancer patients

**DOI:** 10.1038/srep23163

**Published:** 2016-03-17

**Authors:** Sebastian Gnosa, Ivana Ticha, Staffan Haapaniemi, Xiao-Feng Sun

**Affiliations:** 1 Department of Oncology and Department of Clinical and Experimental Medicine, Linköping University, Linköping, Sweden; 2 Institute of Pathology, First Faculty of Medicine, Charles University in Prague and General University Hospital in Prague, Prague, Czech Republic; 3 Department of Surgery and Department of Clinical and Experimental Medicine, Linköping University, Norrköping, Sweden

## Abstract

The colorectal carcinogenesis is a complex process encompassing genetic alterations. The oncoprotein AEG-1, encoded by the *MTDH* gene, was shown previously to be involved in colorectal cancer (CRC). The aim of this study was to determine the frequency and the spectrum of *MTDH* variants in tumor tissue, and their relationship to clinicopathological variables in CRC patients. The study included tumors from 356 unselected CRC patients. Mutation analysis of the *MTDH* gene, including coding region and adjacent intronic sequences, was performed by direct DNA sequencing. The corresponding normal colorectal tissue was analyzed in the carriers of exonic variant to confirm germline or somatic origin. We detected 42 intronic variants, where 25 were novel. Furthermore, we found 8 exonic variants of which four, one missense (c.977C > G-germline) and three frameshift mutations (c.533delA-somatic, c.1340dupA-unknown origin, c.1731delA-unknown origin), were novel. *In silico* prediction analyses suggested four deleterious variants (c.232G > T, c.533delA, c.1340dupA, and c.1731delA). There were no correlations between the *MTDH* variants and tumor stage, differentiation or patient survival. We described several novel exonic and intronic variants of the *MTDH* gene. The detection of likely pathogenic truncating mutations and alterations in functional protein domains indicate their clinical significance, although none of the variants had prognostic potential.

Colorectal cancer (CRC) is the third most common cancer in men and the second in women with 1.36 million incidences per year worldwide. About 700,000 estimated deaths per year caused by CRC making it the fourth most common cause of cancer death, accounting for about 8.5% worldwide^1^. Around 75% of the CRC incidences are sporadic, and the rest of the cases are hereditary or familial CRC, associated with inherited genetic aberrations^2^. As first proposed by Fearon and Vogelstein in 1990, colorectal carcinogenesis is a complex process implicating accumulation of genetic alterations in oncogenes and tumor suppressor genes^3^. Several oncogenic aberrations including point mutations, insertions, deletions and gene amplification in KRAS, NRAS, BRAF, MYC, WNT and PIK3CA have been linked to colorectal carcinogenesis and are therefore promising genetic markers for early cancer detection, treatment selection and prognosis^3^,^4^,^5^.

Current research is devoted to search for new prognostic and predictive biomarkers. The Metadherin gene (MTDH; MIM#610323) encodes for the lysine-rich oncoprotein Astrocyte elevated gene 1 (AEG-1), also called LYRIC, which is highly basic 582 amino acid protein with a molecular mass of 64 kDa^6^,^7^. The gene is located at chromosome 8q22 and comprises 12 (coding) exons and spans around 95 kb (PMID: 14980505)^8^. Amplification of genomic loci 8q22 has been correlated to increased AEG-1 expression^9^,^10^,^11^,^12^,^13^. Several functional regions in the AEG-1 protein have been discovered. The AEG-1 protein contains an N-terminal transmembrane domain (amino acid (aa)51–72), three putative nuclear localization signals (aa79–91, aa432–451 and aa561–580) and several protein interaction sites^14^.

We and others have shown that the AEG-1 mRNA and protein are overexpressed in CRC and other types of cancer compared with the corresponding non-tumor tissue^15^,^16^,^17^,^18^,^19^. The AEG-1 protein has been found to be involved in cell proliferation, survival, migration, invasion, apoptosis, angiogenesis, metastasis and treatment resistance when interacting with a variety of proteins and protein complexes^11^,^13^,^17^,^20^,^21^,^22^,^23^. Two studies conducted on blood samples from breast and ovarian cancer patients have analyzed the coding sequence of *MTDH,* and identified a correlation between the polymorphisms c.1353G > A (rs2331652, p.K451K), and c.1679–6 T > C (rs117026063), and breast cancer susceptibility as well as between the polymorphism −470 G > A and ovarian cancer susceptibility^20^,^24^.

However, it is unknown whether mutations in the *MTDH* gene contribute to tumor progression and have prognostic potential for CRC. The aim of this study was to determine the frequency and the spectrum of *MTDH* variants in tumor tissue and their relationship to clinicopathological variables (patient gender, age at diagnosis, tumor location, tumor stage, grade of differentiation, recurrence and survival) of CRC patients. To our knowledge, this is the first study analyzing mutations of *MTDH* in tumor tissue.

## Results

### Frequency of *MTDH* variants in CRC patients and cell lines

**Figure 1:**
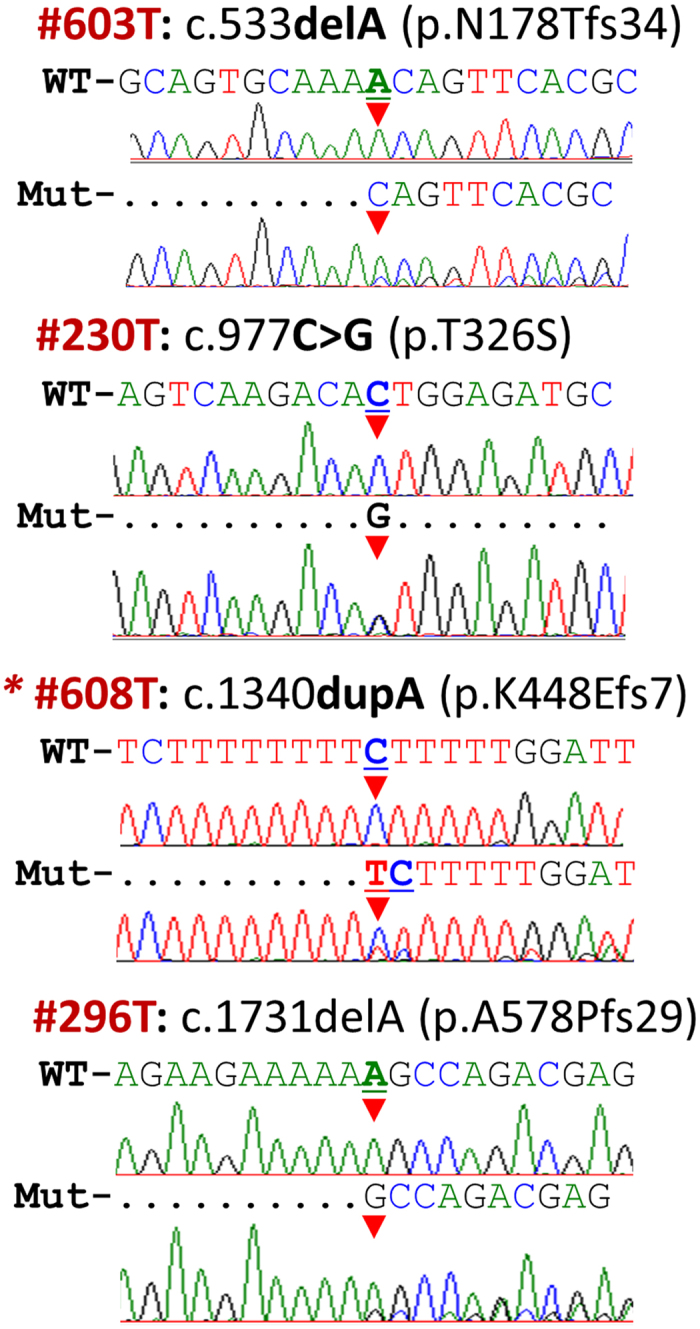
Novel exonic *MTDH* variants. Comparison between wild type sequences and respective samples with mutation for three frameshift variants and one missense variant; wt-wild type sequence, mut–mutated sequence, ^#^identification number of sample, T–tumor tissue. First changed nucleotide is indicated by *red triangle*.

By direct DNA sequencing of the complete coding sequence of the *MTDH* gene, we found 50 single nucleotide variants in 356 CRC patient samples (Supplementary Table 1). Eight of the variants were exonic and 42 were in a non-coding region adjacent to an exon. Among them, there were four novel exonic variants (Table 1, Fig. 1) [c.533delA (p.N178Tfs34), c.977C > G (p.T326S), c.1340dupA (p.K447Efs7) and c.1731delA (p.A578Profs29)], and 25 novel variants in a non-coding region adjacent to exons. All variants found were heterozygous, except for the seven variants c.232G > T, c.382–50C > T, c.568 + 213delT, c.949A > G, c.1048 + 131T > G, c.1049–97delA and c.1147 + 28delT. The genotypic frequency is stated in Supplementary Table 1. There was no *MTDH* variant in the colon cancer cell lines SW480, SW620 and HCT116 (data not shown).

Several variants co-occurred and two clusters were identified (Supplementary Table 2 and 3). The first cluster of variants with a high linkage included the variants c.160G > A (rs140652237, p.V54M), c.568 + 213delT (rs34735761) and c.1353G > A (rs2331652, p.K451K), and showed a significant correlation to each other (p < 0.05). The second cluster of variants with a high linkage included c.232G > T (rs17854373, p.A78S), c.382–50C > T (rs16896067), c.949A > G (rs17854374, p.T317A), c.1048 + 131T > G (rs12675731), c.1049–97delA (rs150495888), and c.1147 + 28delT (rs76537339; p < 0.05). Each variant of both clusters was also detected in the corresponding normal mucosa, which corresponded with their germline origin.

### Intronic *MTDH* variants in relation to clinicopathological variables

The intronic variants c.382–50C > T (rs16896067), c.1048 + 131T > G (rs12675731) and c.1353G > A (rs2331652, p.K451K) were more frequent in the patients <72 years old compared to the age group ≥72 years old (p = 0.019, p = 0.047 and p = 0.021, respectively; Supplementary Table 4). The variant c.1048 + 82 delA (rs149869061) was only detected in tumors located in the colon but not those located in the rectum (p = 0.013). We did not find any relationship between the variants and the gender, tumor stage, grade of differentiation, recurrence and patient survival (p > 0.05).

### Exonic variants in relation to clinicopathological variables and location in functional protein domains

Among the 8 exonic variants detected in this study, four were missense [c.160G > A (rs140652237, p.V54M), c.232G > T (rs17854373, p.A78S), c.949A > G (rs17854374, p.T317A) and c.977C > G, (p.T326S)], one silent [c.1353G > A (rs23316529, p.K451K)], and three frame shift mutations [c.533delA (p.N178Tfs34), c.1340dupA (p.K448Efs7), and c.1731delA (p.A578Pfs29)]. To evaluate whether the exonic variants occurred during colorectal carcinogenesis or whether they are inherited, we analyzed the corresponding normal mucosa of the colon and rectum from the same patients. Frame-shift mutation c.533delA was not detected in the corresponding normal mucosa, and therefore considered as a somatic mutation. The corresponding normal mucosa for the other two frameshift variants was not available, therefore we were not able to assess the somatic or germline status. The other exonic variants were detected also in the corresponding normal mucosa (Table 1). The variant c.232G > T (rs17854373, p.A78S) was more frequent in the patients <72 years old compared to the age group ≥72 years old (p = 0.001; Supplementary Table 4). To evaluate the predicted effects of exonic variants on protein function, six *in silico* prediction tools were used. The *in silico* prediction analyses revealed that four of these variants c.232G > T (rs17854373, p.A78S), c.533delA, c.1340dupA and c.1731delA, were deleterious (Table 1, Fig. 1, Supplementary Table 5). The variants, c.533delA, and c.1340dupA, lead to a truncation of the protein while the variant, c.1731delA, is predicted to lead to protein prolongation. All three variants were heterozygotic and detected in stage I or II colon cancer with moderate or poor differentiation (Table 2).

We discovered two variants which are located in at least one functional region of the AEG-1 protein. The variant c.160G > A (rs140652237, p.V54M), is located in the transmembrane domain and in the CBP and PLZF binding region. The variant, c.232G > T (rs17854373, p.A78S) is located one amino acid before the N-terminal nuclear localization signal and in the YY1, BCCIP and PLZF binding region. The missense variants, c.949A > G (rs17854374, p.T317A) and c.977C > G (p.T326S), are in an area without known protein interaction.

## Discussion

Overexpression of the oncogene AEG-1 has been reported in several types of cancers and was correlated to increased cell proliferation, invasion, survival and treatment resistance^11^,^13^,^17^,^20^,^21^,^22^,^23^. Numerous studies have shown that overexpression of AEG-1 is due to amplification of the genomic loci at chromosome 8q22, activation of up-stream signaling as well as deregulation of several miRNAs^9^,^10^,^11^,^12^,^13^,^25^,^26^,^27^,^28^,^29^,^30^,^31^,^32^. However, it remains largely unclear whether mutations in the *MTDH* gene contribute to its oncogenic properties. In the present study, we therefore examined the frequency and spectrum of *MTDH* variants, and their relationship to clinicopathological variables in 356 CRC patients including tumor tissue as well as in three colon cancer cell lines. In total, we detected 42 intronic variants, whereof 25 were novel. Furthermore, we found eight exonic variants of which four variants, one missense (c.977C > G) and three frameshift mutations (c.533delA, c.1731delA, c.1340dupA), were novel. The three frameshift variants are likely pathogenic.

Correlation analyses between recurrent variants and clinicopathological variables revealed that the intronic variant, c.1048 + 82 delA (rs149869061), was only detected in tumors located in the colon but not those located in the rectum. In a previous study, we found significantly lower expression of the AEG-1 mRNA in the colon compared to the rectum^16^. Whether the intronic variant has an influence on the mRNA expression or stability needs further investigation.

The variants, c.232G > T (rs17854373, p.A78S), c.382–50C > T (rs16896067), c.1048 + 131T > G (rs12675731) and c.1353G > A (rs2331652, p.K451K), were found to be more frequent in the patients <72 years old than those ≥72 years old. However, these variants are hereditary and their impact of the early onset is questionable.

The variants c.1353G > A (rs2331652) and c.1679–6T > C (rs117026063) were both frequently detected in blood samples from breast cancer patients (52% and 22%, respectively) and from healthy controls (36% and 11%, respectively), and both variants have been correlated to breast cancer susceptibility in a Chinese study^24^. Compared to their results, in the present study the variants, c.1353G > A (rs2331652) and c.1679–6T > C (rs117026063), were very rare (2.5% and 0.3%, respectively). The different frequencies in the two studies could be due to the divergence between the ethnical groups (Chinese versus Caucasian), DNA origins and disease mechanisms etc. However, there were no correlations between these two variants and clinicopathological variables, neither in breast cancer^24^ nor in our study.

Several detected exonic variants in this study are located in a functional- or protein binding region of the AEG-1 protein. Even though the three-dimensional structure of AEG-1 is not completely solved, a transmembrane domain, three putative nuclear localization signals as well as several protein interaction regions have been identified^7^,^33^. Variant, c.160G > A (rs140652237, p.V54M), is located in the transmembrane domain which spans the aa51–72 as well as in the CBP and PLZF binding region. Two programs, Polyphen-2 and MUpro, predict this mutation as possibly damaging or lowering stability of the AEG-1 protein. Another variant, c.232G > T (rs17854373, p.A78S), is located one amino acid before the N-terminal nuclear localization signal (aa79–91) and in the YY1, BCCIP and PLZF binding region. Previously, it has been shown that the extended nuclear localization region between aa78–130 regulates the nucleolar localization of AEG-1^33^. Three programs, Mutation Taster, Polyphen-2 and MUpro, predict this mutation to be possibly disease causing or damaging or reducing the protein stability. However, whether these two missense variants have an impact on the protein function has to be experimentally validated.

In conclusion, this is the first study analyzing *MTDH* mutations in tumor tissue. We found 29 novel *MTDH* variants. The three frameshift variants detected in tumor tissue are likely pathogenic, and the other variants detected in functional protein regions suggest their role in CRC tumorigenesis, although none of the variants had prognostic potential. These results suggest that genetic variants of *MTDH* are probably not of high clinical importance in CRC, even though our sample set is relatively small in order to show significance of rare variants.

## Material and Methods

### Patients

This study included primary CRC tissue and distant normal mucosa from 356 CRC patients diagnosed at the University Hospital in Linköping and Vrinnevi Hospital in Norrköping. Tissues were collected during primary surgery between 1989 and 2004. Samples from the corresponding normal tissue of the colon or rectum were taken at least 10 cm from the tumor margins. Representative tumor tissues, evaluated by pathologist, were stored for subsequent analyses at −70 °C. Characteristics of the patients are shown in Table 3. The mean age at diagnosis was 72 years. The tumors with better differentiation included well and moderately differentiated tumors, and worse differentiation included poorly differentiated, mucinous or signet-ring cells carcinomas. Information was lacking about tumor differentiation in four patients and recurrence in 169 patients. The study was approved by the Regional Ethical Review Board in Linköping and an informed consent document was signed by participants. The methods were carried out according to the approved ethical guidelines.

### Cell culture

The SW480 and SW620 cell lines were obtained from American Type Culture Collection.

The cell lines were maintained at 37 °C and 5% CO_2_ in Eagles MEM (Sigma-Aldrich, St. Louis, MO), supplemented with 10% heat inactivated fetal bovine serum albumin (GIBCO, Invitrogen, Paisley, UK) and 1% L-glutamin (GIBCO). The HCT116 cell line was obtained from the Core cell center (Johns Hopkins University, Baltimore, MD) and was maintained in McCoy’s 5A medium (Sigma-Aldrich) supplemented with 10% heat inactivated fetal bovine serum albumin (GIBCO) at 37 °C and 5% CO_2_. Cells growing exponentially were harvested when 80% confluence was achieved. All cells were tested for Mycoplasma by using a commercially available PCR kit (PromoKine, Heidelberg, Germany). The morphology and growth rate of all cell lines were controlled during the whole experimental period.

### Isolation of DNA and mutation analysis

DNA was isolated from fresh frozen tissue and lysate from cell lines using standard procedures implementing DNeasy Blood & Tissue Kit (Qiagen, Hilden, Germany). The coding region of the *MTDH* gene was analyzed by using PCR and direct DNA Sanger sequencing in 356 tumors. The exons 1 to 12 and adjacent intronic sequences were amplified using FastStart High Fidelity PCR System (Roche Applied Science, Germany) according to the manufacturer’s instructions. BigDye Terminator *v*3.1 Ready Reaction Mix (Applied Biosystems, Foster City, CA) was used for sequencing reaction, and separation was performed on ABI 3500 genetic analyzer (Applied Biosystems). The collected data were analyzed by using Sequence analyzer software (Applied Biosystems). Designed primers used for amplification and sequencing analysis are shown in Table 4. Each variant or suspicious fragment was verified by independent PCR amplification and sequence analysis in tumor. Exonic variants that were detected in tumor tissue were analyzed also in the corresponding normal tissue (when available) from the same patients. All detected variants were confirmed by sequencing of forward and reverse strands.

### Nomenclature of mutations

Mutations were described according to the nomenclature system recommended by the Human Genome Variation Society (HGVS)^34^. Designation of the genomic alterations in the *MTDH* gene is based on the GenBank reference sequences NM_178812. Mutations which were not found in the literature, the Single Nucleotide Polymorphism Database (dbSNP, http://www.ncbi.nlm.nih.gov/SNP/, (accessed in June, 2015)^35^, or in the Catalogue of Somatic Mutations in Cancer (COSMIC, http://www.sanger.ac.uk/cosmic, accessed in June, 2015)^36^ were considered as novel.

### Statistical analyses

Importance of frequent variants was analyzed by using the STATISTICA 10 (StatSoft, Tulsa, OK). The chi-square test was applied to determine the relationship of *MTDH* variants with clinicopathological variables. Cox’s Proportional Hazard Model was used to test the relationship between the variants and the patient survival. All tests were two sided, and a *P*-value less than 0.05 was considered as significant.

### *In silico* prediction of impact of the variants on protein function

Exonic variants were evaluated by widely used programs for prediction of possible interference with the function, structure or stability of a protein (Supplementary Table 5): Mutation Taster (http://www.mutationtaster.org; Ensembl transcript ENST00000336273, NM_178812; GRCh37/ Ensembl 69), SIFT and GVGD as a part of commercial Alamut 2.0 (Interactive Biosoftware, Roven, France), PolyPhen-2 (http://genetics.bwh.harvard.edu/pph2/; UniProt peptide Q86UE4), PROVEAN (http://provean.jcvi.org/index.php; Human GRCh37/Ensemble 66) and, MUpro (http://mupro.proteomics.ics.uci.edu).

## Additional Information

**How to cite this article**: Gnosa, S. *et al*. *MTDH* genetic variants in colorectal cancer patients. *Sci. Rep.* **6**, 23163; doi: 10.1038/srep23163 (2016).

## Supplementary Material

Supplementary Information

## Figures and Tables

**Table 1:** Exonic variants detected in the *MTDH* gene in colorectal cancer patients.

Exon	cDNA^a^	n (%)	Reference^b^	Predictedmutation effect	*in silico*prediction^c^	Origin^d^
1	c.160G > A	4 (1.1)	rs140652237	p.V54M	polymorphism	germline
1	c.232G > T	35 (10)	rs17854373	p.A78S	pathogenic	germline
3	**c.533delA**	1 (0.3)	novel	p.N178Tfs34	pathogenic	somatic
6	c.949A > G	56 (16)	rs17854374	p.T317A	polymorphism	germline
6	c.977C > G	1 (0.3)	novel	p.T326S	polymorphism	germline
9	**c.1340dupA**	1 (0.3)	novel	p.K448Efs7	pathogenic	N/A
9	c.1353G > A	9 (2.5)	rs2331652	p.K451K	polymorphism	germline
12	**c.1731delA**	1 (0.3)	novel	p.A578Pfs29	pathogenic	N/A

^*a*^GenBank reference sequence NM_178812 (7667bp mRNA): +1 corresponds to the A of the ATG translation initiation codon.

^*b*^dbSNPdatabase.

^*c*^as pathogenic are denominated frameshift variants or variants predicted pathogenic by at least 2 predictive programs; frame-shift variants are indicated in bold.

^*d*^variants were considered as somatic if they were not detected in corresponding normal mucosa, otherwise they were considered germline; N/A normal tissue was not available.

**Table 2:** Exonic *MTDH* variants in relation to clinicopathological variables of colorectal cancer patients.

Characteristics	c.160G > Ap.V54Mrs140652237	c.232G > Tp.A78Srs17854373het/ho	c.533delAp.N178Tfs34novel	c.949A > Gp.T317Ars17854374het/ho	c.977C > Gp.T326Snovel	c.1340dupAp.K448Efs7novel	c.1353G > Ap.K451Krs2331652	c.1731delAp.A578Pfs29novel
Gender								
Male	2	18/2	1	28/2	0	1	4	0
Female	2	14/1	0	25/1	1	0	5	1
Age at diagnosis (mean)								
<72 years	3	21/2	0	27/2	1	0	7	1
≥72 years	1	11/1	1	26/1	0	1	2	0
Tumor location								
Colon	2	20/2	1	29/2	0	1	5	1
Rectum	2	12/1	0	24/1	1	0	4	0
Tumor stage								
I	0	2/0	0	4/0	1	1	0	0
II	1	14/2	1	23/2	0	0	4	1
III	3	12/1	0	20/1	0	0	4	0
IV	0	4/0	0	6/0	0	0	1	0
Differentiation^a^								
Well	1	2/0	0	6/0	0	0	2	0
Moderately	3	19/2	0	32/2	1	1	5	0
Poorly	0	11/1	1	15/1	0	0	2	1

^a^Data not available for some patients.

**Table 3:** Colorectal cancer patients and tumor characteristics.

Characteristics	356 CRC tumors (%)
Gender	
Male	190 (53)
Female	166 (47)
Age at diagnosis (mean)	
<72 years	144 (40)
≥72 years	212 (60)
Patient survival (mean)^a^	
<70 months	216 (61)
≥70 months	140 (39)
Tumor location	
Colon	203 (57)
Rectum	153 (43)
Tumor stage	
I	44 (12)
II	150 (42)
III	108 (30)
IV	54 (15)
Differentiation^b^	
Well	32 (9)
Moderately	222 (63)
Poorly	98 (28)
Recurrence^a^	
Yes	137 (73)
No	50 (27)

^a^median survival is 50 months.

^b^data not available for some patients.

**Table 4:** Primer pairs used for PCR amplification and sequence analysis of the *MTDH* gene.

Exon	Primers 5′ → 3′	Length of PCR product (bp)
1	F: ACCAATTAACCCCTCCCAGC	1087
	R: CCTCTCGGCTTTCGACTAAG	
	SqF1^b^: TTCCCTGACACGCCTTTG	
	SqF2: TCGCTTCCCTCGACTATTCC	
2	F: AGGTACAGAGGTAGGATTTG	556
	R: AAGGTAACACAATTCCACAG	
3–4	F: TTGTCAGCATCATACATTTC	1183
	R: ACATGGTTCACAATACTCTC	
	SqR1: AACCATAATTCCAGGAGAC	
	SqF2: TCAACACTCTTGGTTTTAGC	
5	F: GTGGAAGAATTTAGCACTTG	396
	R: ATCGTTAGAAGTGGGTGTG	
6	F: TAAGGCAATCCTTGGTGATC	547
	R: AATTCCCACCTTGCTCTAC	
7	F: ATCTAATGGATTGGTGCTAGG	642
	R: TAGGAGGAGAAACAGACATTC	
8	F: TGGCTCTTAAAATGTGCTTGG	846
	R: ATTGGTGTCAGCCTCTGTG	
9	F: ATGACGTAGACACACTGAGAG	720
	R: ACCAGCAAACTCAAAGTCAAG	
10	F: AGCAATTCTCATACCTCCTC	677
	R: TGCTCTTGAACTCCTGACCT	
	SqF: TCTCTCAGGCTCAAGTAGTCC	
11	F: AGAGGGCAGGTATGAGTTAC	477
	R: TAGCCAGGATGGTCTTGATG	
12	F: AAGGAGGGAAGAAGACATAG	469
	R: TTCCCAAGTGTCTTCCATC	

^*a*^GenBank reference sequence NC_000008 (chr8:98,656,407–98,742,488; GRCh37).

^*b*^underlined primers were preferentially used for sequence analysis.
