# Supplementary material for: MTDH genetic variants in colorectal cancer patients
Source: Sci Rep. 2016 Mar 17;6:23163. doi: 10.1038/srep23163 (PMC4794727; doi:10.1038/srep23163)
Supplement: Supplementary Information [file srep23163-s1.doc]

***MTDH* genetic variants in colorectal cancer patients**

Sebastian Gnosa, Ivana Ticha, Staffan Haapaniemi, Xiao-Feng Sun

Supplementary Table 1. All variants detected in the *MTDH* gene

in tumor tissue of colorectal cancer patients.

| Exon/ | cDNA*^a^* | Frequency | Reference*^b^* |
| --- | --- | --- | --- |
| Intron | Predicted effect | n (%) |  |
| 5´UTR | c.1-328-31C>A | 1 (0.3) | novel |
| 5´UTR | c.1-328-1C>T | 7 (2) | rs572908844 |
| 5´UTR | c.1-321G>C | 2 (0.6) | rs565176281 |
| 5´UTR | c.1-212G>A | 1 (0.3) | novel |
| 5´UTR | c.1-305C>G | 17 (5) | rs116862822 |
| Exon 1*^c^* | c.160G>A  p.V54M | 4 (1.1) | rs140652237 |
| Exon 1 *^d^* | c.232G>T  p.A78S |  | rs17854373 |
|  | G/G | 321 (90) |  |
|  | G/T | 32 (9) |  |
|  | T/T | 3 (1) |  |
| Intron 1 | c.381+57C>T | 1 (0.3) | rs550432304 |
| Intron 1*^d^* | c.382-50C>T |  | rs16896067 |
|  | C/C | 207 (58) |  |
|  | C/T | 125 (35) |  |
|  | T/T | 24 (6.7) |  |
| Intron 2 | c.483+179dubT | 2 (0.6) | novel |
| Exon 3 | c.533delA  p.N178Tfs34 | 1 (0.3) | novel |
| Intron 3 | c.568+109T>G | 1 (0.3) | novel |
| Intron 3 | c.568+113dupT | 1 (0.3) | novel |
| Intron 3 | c.568+159C>T | 1 (0.3) | novel |
| Intron 3 *^c^* | c.568+213delT |  | rs34735761 |
|  | T/T | 329 (92.1) |  |
|  | T/- | 24 (6.7) |  |
|  | -/- | 3 (1) |  |
| Intron 3 | c.568+294delT | 1 (0.3) | novel |
| Intron 3 | c569-16dupT | 1 (0.3) | novel |
| Intron 3 | c569-16delT | 2 (0.6) | novel |
| Intron 5 | c.811+61A>G | 1 (0.3) | rs78603958 |
| Intron 5 | c.811+101delA | 1 (0.3) | novel |
| Intron 5 | c.811+168A>G | 2 (0.6) | novel |
| Intron 5 | c.812-12C>A | 1 (0.3) | novel |
| Exon 6 *^d^* | c.949A>G  p.T317A |  | rs17854374 |
|  | A/A | 300 (84) |  |
|  | A/G | 53 (14.8) |  |
|  | G/G | 3 (1) |  |
| Exon 6 | c.977C>G  p.T326S | 1 (0.3) | novel |
| Intron 6 | c.1048+38T>A | 2 (0.6) | rs370107758 |
| Intron 6 | c.1048+57delT | 1 (0.3) | novel |
| Intron 6 | c.1048+82delA | 8 (2.3) | rs149869061 |
| Intron 6 *^d^* | c.1048+131T>G |  | rs12675731 |
|  | T/T | 203 (56.9) |  |
|  | T/G | 130 (36.5) |  |
|  | G/G | 23 (6.5) |  |
| Intron 6 | c.1049-158C>A | 1 (0.3) | novel |
| Intron 6 *^d^* | c.1049-97delA |  | rs150495888 |
|  | A/A | 298 (83.4) |  |
|  | A/- | 55 (15.4) |  |
|  | -/- | 3 (1) |  |
| Intron 6 | c.1049-10T>C | 1 (0.3) | novel |
| Intron 7 *^d^* | c.1147+28delT |  | rs76537339 |
|  | T/T | 204 (57.1) |  |
|  | T/- | 133 (37.2) |  |
|  | -/- | 19 (5.3) |  |
| Intron 7 | c.1147+35dupT | 1 (0.3) | novel |
| Intron 8 | c.1272+145A>G | 3 (1) | rs181944409 |
| Exon 9 | c.1340dupA  p.K447fsx7 | 1 (0.3) | novel |
| Exon 9 *^c^* | c.1353G>A  p.K451K | 9 (2.5) | rs2331652 |
| Intron 9 | c.1380+104G>A | 1 (0.3) | novel |
| Intron 9 | c.1380+250T>A | 2 (0.6) | novel |
| Intron 9 | c.1380+250delT | 1 (0.3) | novel |
| Intron 9 | c.1381-186A>T | 1 (0.3) | novel |
| Intron 10 | c.1521+16A>G | 2 (0.6) | rs202138651 |
| Intron 10 | c.1521+51A>G | 1 (0.3) | novel |
| Intron 10 | c.1522-11delT | 1 (0.3) | novel |
| Intron 11 | c.1678+129G>T | 1 (0.3) | rs556608672 |
| Intron 11 | c.1679-59G>A | 1 (0.3) | novel |
| Intron 11 | c.1679-28delT | 1 (0.3) | novel |
| Intron 11 | c.1679-6T>C | 1 (0.3) | rs117026063 |
| Intron 11 | c.1679-24C>T | 1 (0.3) | rs544391839 |
| Exon 12 | c.1731delA  p.A578Profs*29 | 1 (0.3) | novel |
| 3´UTR | c.1749+9delT; c.*+10 delT | 1 (0.3) | novel |

*^a^*GenBank reference sequence NM_178812 (7667bp mRNA): +1 corresponds to the A of the ATG translation initiation codon; *^b^* dbSNPdatabase; *^c^* co-occurred variants cluster 1; *^d^* co-occurred variants cluster 1.

Supplementary Table 2. Haplotypes of co-occurring variants (cluster 1) in the colorectal cancer patients.

|  | Variant  c.160G>A | Variant  c.568+213delT | Variant  c.1353G>A | Frequency  n (%) |
| --- | --- | --- | --- | --- |
|  | **G/G** | **T/T** | **G/G** | 324 (91) |
|  | **G/G** | T/- | **G/G** | 20 (5.6) |
|  | **G/G** | **T/T** | G/A | 4 (1.1) |
|  | **G/G** | -/- | **G/G** | 3 (0.8) |
|  | G/A | T/- | G/A | 3 (0.8) |
|  | **G/G** | T/- | G/A | 1 (0.3) |
|  | G/A | **T/T** | G/A | 1 (0.3) |

Wild type variants in **bold**

Supplementary Table 3. Haplotypes of co-occurring variants (cluster 2) in the colorectal cancer patients.

|  | **Variant**  **c.232G>T** | **Variant**  **c.382-50C>T** | | **Variant**  **c.949A>G** | | **Variant**  **c.1048+131T>G** | | **Variant**  **c.1049-97delA** | | **Variant**  **c.1147+28delT** | | **Frequency**  **n (%)** | |
| --- | --- | --- | --- | --- | --- | --- | --- | --- | --- | --- | --- | --- | --- |
|  | **G/G** | **C/C** | **A/A** | | **T/T** | | **A/A** | | **T/T** | | 196 (55.1) | |  |
|  | **G/G** | C/T | **A/A** | | T/G | | **A/A** | | T/- | | 79 (22.2) | |  |
|  | G/T | C/T | A/G | | T/G | | A/- | | T/- | | 23 (6.5) | |  |
|  | **G/G** | C/T | A/G | | T/G | | A/- | | T/- | | 18 (5.1) | |  |
|  | **G/G** | T/T | **A/A** | | T/G | | **A/A** | | -/- | | 9 (2.5) | |  |
|  | **G/G** | **C/C** | **A/A** | | T/G | | **A/A** | | T/- | | 5 (1.4) | |  |
|  | G/T | T/T | A/G | | G/G | | A/- | | **T/T** | | 4 (1.1) | |  |
|  | G/T | T/T | A/G | | G/G | | A/- | | -/- | | 3 (0.8) | |  |
|  | T/T | T/T | A/A | | G/G | | -/- | | -/- | | 3 (0.8) | |  |
|  | **G/G** | **C/C** | **A/A** | | **T/T** | | **A/A** | | T/- | | 2 (0.6) | |  |
|  | **G/G** | C/T | **A/A** | | **T/T** | | **A/A** | | **T/T** | | 2 (0.6) | |  |
|  | **G/G** | T/T | A/G | | G/G | | A/- | | -/- | | 2 (0.6) | |  |
|  | **G/G** | **C/C** | **A/A** | | **T/T** | | A/- | | T/- | | 1 (0.3) | |  |
|  | **G/G** | **C/C** | A/G | | T/G | | A/- | | T/- | | 1 (0.3) | |  |
|  | **G/G** | **C/C** | A/G | | G/G | | A/- | | -/- | | 1 (0.3) | |  |
|  | **G/G** | C/T | **A/A** | | **T/T** | | **A/A** | | T/- | | 1 (0.3) | |  |
|  | **G/G** | C/T | **A/A** | | T/G | | A/- | | T/- | | 1 (0.3) | |  |
|  | **G/G** | T/T | **A/A** | | T/G | | **A/A** | | T/- | | 1 (0.3) | |  |
|  | **G/G** | T/T | **A/A** | | T/G | | **A/A** | | -/- | | 1 (0.3) | |  |
|  | **G/G** | T/T | A/G | | G/G | | A/- | | **T/T** | | 1 (0.3) | |  |
|  | G/T | **C/C** | **A/A** | | **T/T** | | **A/A** | | **T/T** | | 1 (0.3) | |  |
|  | G/T | C/T | **A/A** | | T/G | | **A/A** | | T/- | | 1 (0.3) | |  |

Wild type variants in **bold**

Supplementary Table 4. Significant relationship between the *MTDH* variants and the age of colorectal cancer patients.

| Variant | Age | | p-value |
| --- | --- | --- | --- |
|  | < 72 years (%) | ≥ 72 years (%) |  |
| c.232G>T |  |  | 0.001 |
| G/G | 121 (84) | 200 (94) |  |
| G/C & C/C | 23 (16) | 11 (6) |  |
| c.382-50C>T |  |  |  |
| C/C | 73 (51) | 134 (63) | 0.019 |
| C/T & T/T | 71 (49) | 78 (37) |  |
| c.1048+131T>G |  |  | 0.047 |
| G/G | 73 (51) | 130 (61) |  |
| G/T & T/T | 71 (49) | 82 (39) |  |
| c.1353G>A |  |  | 0.021 |
| G/G | 137 (95) | 210 (99) |  |
| G/A & A/A | 7 (5) | 2 (1) |  |

Supplementary Table 5. *In silico* prediction of the impact of the exonic *MTDH* variants on the protein function.

| Exon | cDNA*^a^*  dbSNP*^b^*  n (%) | Predicted mutation effect | Mutation Taster | SIFT  (within Alamut) | GVGD (within Alamut) | PROVEAN | Polyphen-2 | MUpro |
| --- | --- | --- | --- | --- | --- | --- | --- | --- |
| 1 | *^a^*c.160G>A, *^b^*rs140652237  4 (1.1) | p.V54M | Polymorphism | Tolerated | Class C0 | Neutral | Possibly damaging  0.815 | Decrease -0.93 |
| 1 | c.232G>T  rs17854373  35 (10) | p.A78S | Disease causing | Tolerated | Class C0 | Neutral | Possibly damaging  0.995 | Decrease -1 |
| 3 | **c.533delA**  -  1 (0.3) | p.N178Tfs34  210aa (wt 582) | Disease causing | NA/fs | NA/fs | NA/fs | NA/fs | NA/fs |
| 6 | c.949A>G  rs17854374  56 (16) | p.T317A | Polymorphism | Tolerated | Class C0 | Neutral | Benign | Decrease -1 |
| 6 | c.977C>G  -  1 (0.3) | p.T326S | Polymorphism | Tolerated | Class C0 | Neutral | Benign | Decrease -0.28 |
| 9 | **c.1340dupA**  -  1 (0.3) | p.K448Efs7  453aa (wt 582) | Disease causing | NA/fs | NA/fs | NA/fs | NA/fs | NA/fs |
| 9 | c.1353G>A  rs2331652  9 (2.5) | p.K451K | Polymorphism | NA | NA | Neutral | NA | NA |
| 12 | **c.1731delA**  -  1 (0.3) | p.A578Pfs29  605aa (wt 582) | Polymorphism | NA/fs | NA/fs | NA/fs | NA/fs | NA/fs |

*^a^*GenBank reference sequence NM_178812 (7667bp mRNA): +1 corresponds to the A of the ATG translation initiation codon; *^b^* rs# is the reference number from dbSNP Database;

frame-shift variants are indicated in **bold.**
